# EXTENSIVE CUTANEOUS MANIFESTATIONS: PRESENTING FEATURE OF CHRONIC MYELOCYTIC LEUKEMIA IN SECOND BLAST CRISIS

**DOI:** 10.4103/0019-5154.70682

**Published:** 2010

**Authors:** Manish Singhal, Sarika Singh, Rajive Kumar, Vinod Raina

**Affiliations:** *From the Department Medical Oncology, IRCH, AIIMS. All India Institute of Medical Sciences (AIIMS), New Delhi, India*

**Keywords:** *CML*, *Leukemia cutis*, *Blast crisis*

## Abstract

Leukemia cutis is the infiltration of neoplastic leukocytes or their precursors into the epidermis, the dermis, or the subcutis, resulting in clinically identifiable cutaneous lesions. We describe a case of CML who presented with extensive cutaneous manifestations at the time of second blast crisis with multiple subcutaneous skin nodules over the face and trunk with extensive violaceous papules and plaques over all four limbs and the trunk, with scalp showing extensive crusting and scaling with foul smelling discharge.

## Introduction

Leukemia cutis is the infiltration of neoplastic leukocytes or their precursors into the epidermis, the dermis, or the subcutis, resulting in clinically identifiable cutaneous lesions. Leukemia cutis usually presents concomitantly with systemic leukemia or after leukemia has been diagnosed. Less commonly, it may occur as a sole presenting feature in absence of simultaneous marrow involvement.

## Case Report

A 38 year old male was diagnosed to have chronic phase chronic myelocytic leukemia 5 years back.

**Figure 1:**
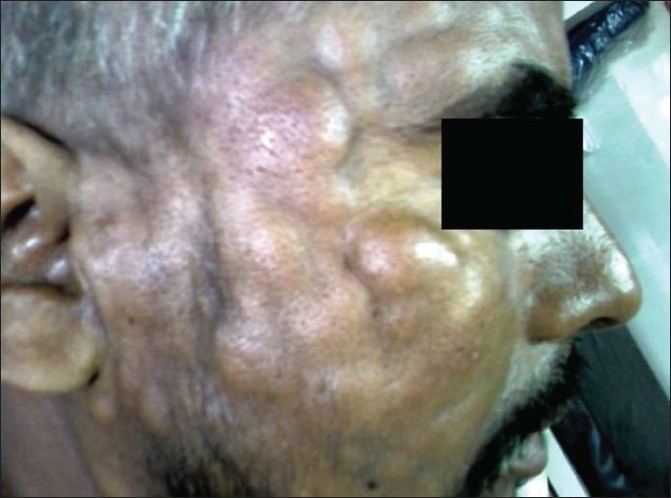
Multiple subcutaneous skin nodules seen over the face

**Figure 2:**
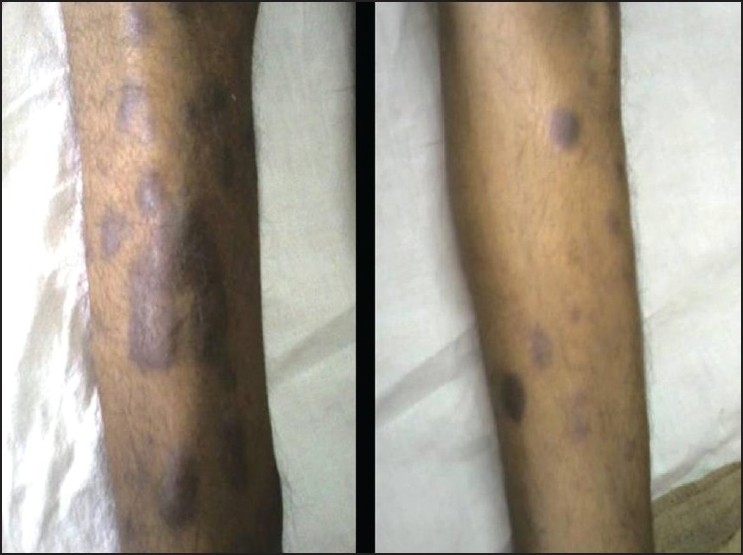
Violaceous papules and plaques seen over the lower limbs

**Figure 3:**
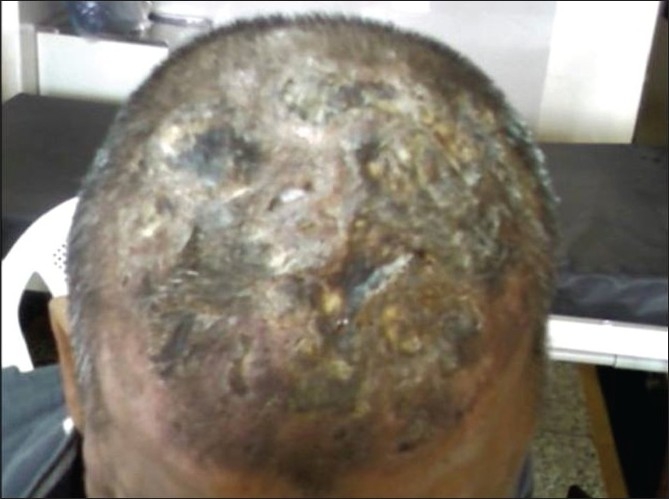
Extensive crusting and scaling seen over the scalp

**Figure 4:**
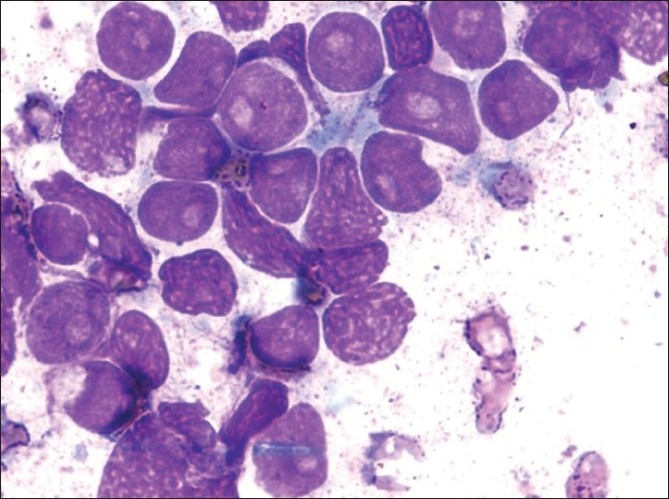
Aspirate from skin nodule showing leukemic cells with lymphoblastoid morphology Giemsa ×40

**Figure 5:**
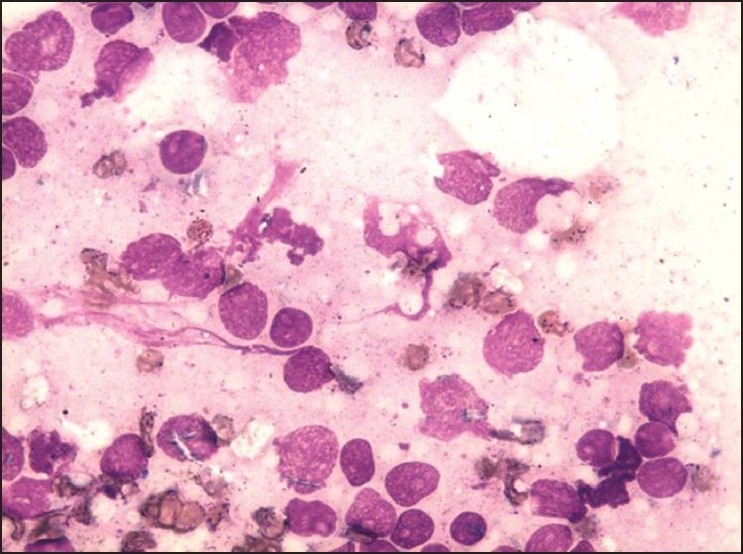
Bone marrow showing extensive blast MGG (May-Grunwald Giemsa) 20×

He was started on imatinib 400 mg per day. He achieved a rapid and complete hematological response and was found to be in complete cytogenetic remission 20 months from the start of therapy. He kept well for another 24 months when he was detected to be losing hematological and cytogenetic response. The dose of imatinib was increased to 600 mg per day, however he progressed rapidly to blast crisis with peripheral smear showing 65% blast and bone marrow being near totally replaced by blast. Immunophenotyping of the blast revealed B cell lymphoid blast crisis (CD19 positive). He was started on acute lymphoblastic leukemia like induction regimen. Post induction he achieved a complete remission and was planned for allogenic stem cell transplant with his HLA identical sister. However, he declined transplant option and continued on imatinib 400 mg per day. He presented 2 months later with fever, weakness, left sided lower motor neuron facial palsy and multiple subcutaneous skin nodules over the face and trunk with extensive violaceous papules and plaques over all four limbs and the trunk [Figures 1 and 2]. His scalp showed extensive crusting and scaling with foul smelling discharge [Figure 3]. He was thought to have extensive leukemic infiltration of the skin. This was confirmed by fine needle aspiration from the skin nodules which showed presence of blast with lymphoblastoid morphology [Figure 4]. His peripheral smear and bone marrow also showed extensive blast [Figure 5], suggestive of a second blast crisis. He was given only supportive therapy this time, which resulted in modest improvement in his general condition. He was not thought to be fit for more aggressive therapy and was referred to palliative care.

## Discussion

Leukemia cutis (LC) is a relatively rare condition and may manifest in a variety of leukemia subtypes. While acute monocytic, myelomonocytic, and the T cell leukemias show the highest incidence (50 to 70%) of LC, it is an uncommon manifestation of chronic myelocytic leukemia (2 to 8%), and when present points towards blastic transformation of the disease.[1,2] Although skin infiltrates have been reported in chronic phase of the disease, this either precedes or occur simultaneously with a blood picture of more aggressive phase, hence the incidence perhaps largely correspond to either the accelerated phase or the blast phase of the disease.[2,3] It is noteworthy that as many as 90% of patients with LC may also have other extramedullary involvement, and as many as 40% may have meningeal or central nervous system involvement.[4]

Leukemia cutis when present as the sole presenting feature of the disease, is described as aleukemic leukemia cutis.[5] In such cases in particular, cytochemistry with myeloperoxidase, naphthol ASD, chloroacetate esterase; immunohistochemical analysis using a panel of antibodies (LCA, CD 3, CD4, CD 5, CD8, CD13, CD19, CD33, CD43, CD45, CD56, CD68), in addition to cytogenetic [t (9:22), etc] and molecular analysis (RT-PCR for bcr-abl) of cells derived from the skin lesions can help establish the primary diagnosis.[2,3,5]

The pathogenesis of LC is not well defined. Chemokine receptors such as CCR4 and adhesion molecules such as cutaneous lymphocyte associated antigen (CLA) have been found to play important role in skin tropism of leukemic cells.[2] The skin lesions in LC show varied morphology and can be difficult to distinguish both clinically and histopathologically from nonspecific cutaneous lesions, which occur much more frequently.[6] Lesions of LC are typically papules and nodules; indurated or hemorrhagic plaques, perifollicular acneiform papules, bullae, and palpable purpura. The lesions can range from red-brown to violaceous or plum color.[2,3] Other unusual manifestations include erythema nodosum, erythema annulare centrifugum, pyoderma gangrenosum, lesions mimicking urticaria, guttate psoriasis, vitiligo, stasis dermatitis and subungual leukemia cutis.[3,7,8] LC may also occur within established scars and within recent areas of trauma.[9] It is important to bear in mind the varied clinical presentations of LC so as to facilitate its early diagnosis and timely treatment. Also, it is important to differentiate LC from sweet syndrome which is an acute febrile neutrophilic dermatosis found in patients of hematological malignancies and present as tender, erythematous, well demarcated papules and plaques and is successfully managed with steroids.[10]

In general, LC is a poor prognostic sign. Several studies indicate that the disease course is aggressive and the length of survival short when CML or AML present with leukemia cutis.

A study by Kaddu *et al*. showed an average survival time of 9.4 months in CML with skin infiltrates.[3] Induction and consolidation chemotherapy followed by allogeneic bone marrow transplant can produce long survival and possibly cure.[1,5]
